# Taste Receptor Polymorphisms and Immune Response: A Review of Receptor Genotypic-Phenotypic Variations and Their Relevance to Chronic Rhinosinusitis

**DOI:** 10.3389/fcimb.2018.00064

**Published:** 2018-03-07

**Authors:** Vasiliki Triantafillou, Alan D. Workman, Michael A. Kohanski, Noam A. Cohen

**Affiliations:** ^1^Department of Otorhinolaryngology–Head and Neck Surgery, University of Pennsylvania, Philadelphia, PA, United States; ^2^Department of Otorhinolaryngology-Head and Neck Surgery, Philadelphia Veterans Affairs Medical Center, Philadelphia, PA, United States

**Keywords:** chronic rhinosinusitis, upper airway immunity, bitter taste receptors, sweet taste receptors, host-pathogen interactions

## Abstract

Bitter (T2R) and sweet taste (T1R) receptors have emerged as regulators of upper airway immune responses. Genetic variation of these taste receptors additionally confers susceptibility to infection and has been implicated in severity of disease in chronic rhinosinusitis (CRS). Ongoing taste receptor research has identified a variety of biologically active compounds that activate T1R and T2R receptors, increasing our understanding of not only additional receptor isoforms and their function but also how receptor function may contribute to the pathophysiology of CRS. This review will discuss the function of taste receptors in mediating airway immunity with a focus on recently described modulators of receptor function and directions for future research into the potential role of genotypic and phenotypic receptor variation as a predictor of airway disease and response to therapy.

## Introduction

The upper respiratory tract depends upon several mechanisms to detect and then mount defenses against pathogenic bacteria to prevent colonization and infection. Failure of these mechanisms is observed in many upper respiratory diseases, including chronic rhinosinusitis (CRS). While the etiology of CRS is multifactorial, defective mucociliary clearance resulting in the stasis of sinonasal secretions is a common final pathophysiologic contributor to the disease. Leading to ultimate mucociliary dysfunction are varied environmental and genetic factors (Antunes et al., 2009).

CRS affects approximately 16% of Americans with costs exceeding $8 billion annually (Bhattacharyya, 2011; Orlandi et al., 2016; Rudmik, 2017). Individuals with CRS often require prolonged antibiotic treatment, with CRS accounting for 20% of adult antibiotic prescriptions (Bhattacharyya and Kepnes, 2008). Patients not only report poor quality of life (QOL) on sinus-specific scales (Gliklich and Metson, 1995; Hopkins et al., 2009), but also more severe impairment in general quality of life (QOL) scores than patients suffering from chronic respiratory or heart diseases (Soler et al., 2011). Despite this enormous economic, quality of life, and public health burden, the pathophysiological mechanisms underlying the disease, including the genetic predisposition to the development of CRS (Cohen et al., 2006; Hamilos, 2007; Fokkens et al., 2012; Adappa et al., 2013; Lee and Cohen, 2013; Cohen, 2017), are just beginning to be elucidated.

Studies in recent years have described an emerging role for bitter and sweet taste receptors as upper airway sentinels that sense secreted bacterial products and subsequently regulate innate immune responses (Tizzano et al., 2010; Lee et al., 2012, 2014). Furthermore, polymorphisms in these receptors contribute not only to individual preferences in taste (Hayes et al., 2011), but also correlate with CRS disease severity (Lee et al., 2012; Adappa et al., 2016b; Carey et al., 2017). This review will discuss the function of taste receptors in mediating airway immunity with a focus on modulators of receptor function and the clinical implications of genotypic and phenotypic variations as predictors of airway disease and response to therapy.

## Mechanisms of sinonasal innate immunity

The sinonasal cavity is the respiratory system's first line of defense against the continuous insult of inhaled pathogens and particulate debris. The sinonasal tract consists of goblet cells which produce a mucus gel and ciliated cells that function not only as a physical epithelial cell barrier but also in mucus transport, clearing debris and microbes trapped in mucus secretions through the process of mucociliary clearance (MCC) (Sleigh et al., 1988). MCC requires coordinated ciliary beating to propel mucus toward the oropharynx where it is swallowed or expectorated and is influenced by mucus viscosity (Liu et al., 2014) as well as mechanical and biological stimuli that accelerate ciliary beating (Shaari et al., 2006; Chen et al., 2007; Zhao et al., 2012; Workman and Cohen, 2014). In addition to their role in MCC, ciliated epithelial cells further function in innate immunity as the source of antimicrobial compounds including β-defensins, lysozyme, lactoferrin, and reactive oxygen species and also recruit the adaptive immune system through cytokine release (Parker and Prince, 2011; Waterer, 2012; Lee et al., 2014).

Initiation of this robust innate and adaptive immune response is coordinated through recognition of microbial products by a variety of receptors. Toll-like receptors (TLRs) are expressed on ciliated airway epithelial cells and recognize conserved bacterial structures called pathogen-associated molecular patterns (PAMPs). Well-known PAMPs include lipopolysaccharide, a major component of the outer membrane of gram-negative bacteria, lipoteichoic acid in gram-positive bacteria, and flagellin (Ooi et al., 2008). TLR activation generates a sustained immune response through changes in gene expression over a period of hours (Hume et al., 2001). Other defensive responses, such as the secretion of antimicrobial compounds and changes in ciliary beat frequency described above occur on a much more rapid timescale (Barham et al., 2013). This suggests that a subset of the upper airway immune response may be initiated through the detection of bacterial products through a non-TLR alternative mechanism.

## Taste receptors regulate upper airway immunity

**Figure 1:**
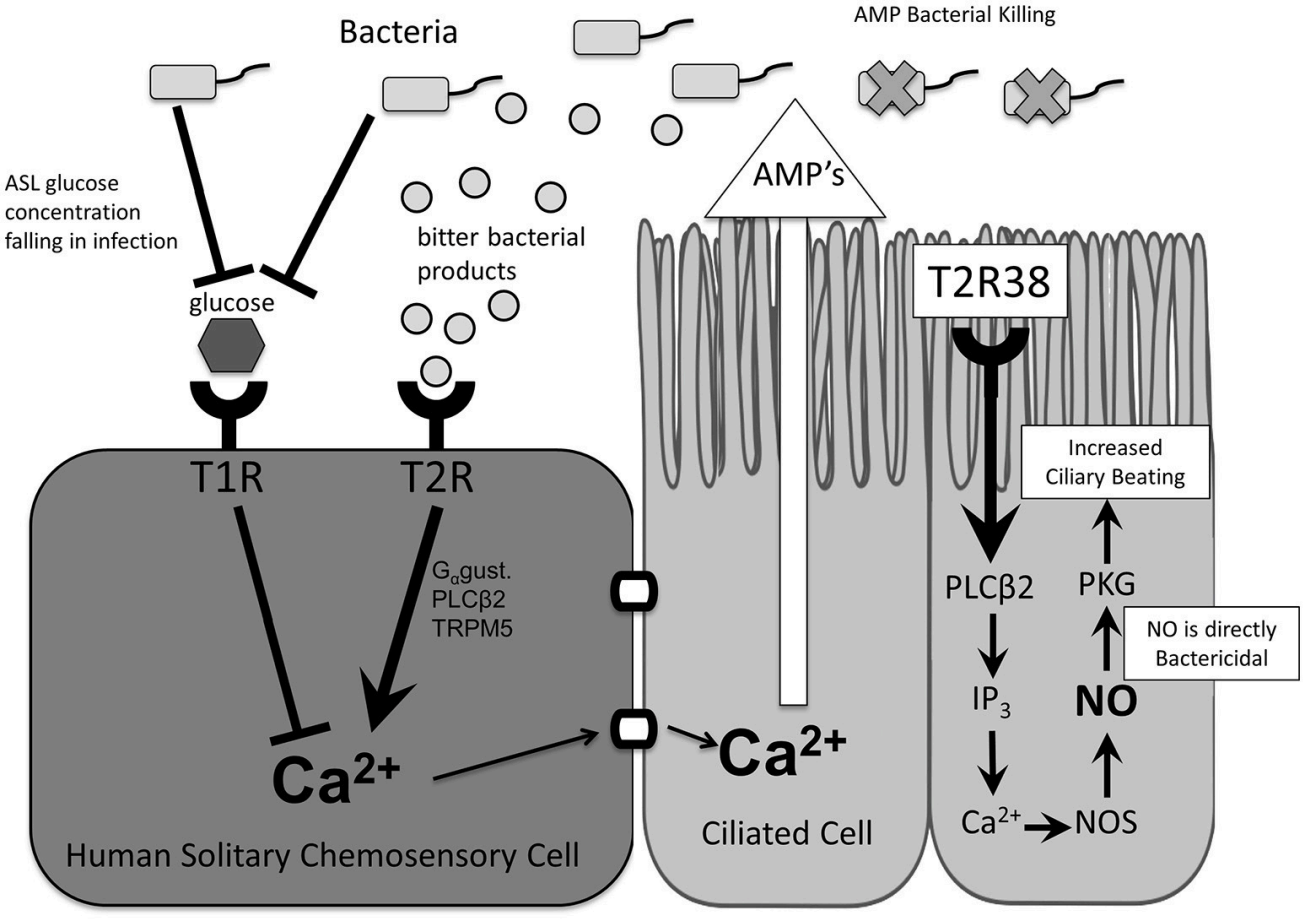
Taste receptor function and downstream effects. In ciliated cells, stimulation of T2R38 and taste-receptor signaling results in activation of innate immune mechanisms including increased mucociliary clearance and nitric oxide release. In solitary chemosensory cells, stimulation of T2Rs results in a calcium-mediated release of antimicrobial peptides. This response is regulated by T1Rs such that under physiologic airway surface liquid glucose levels the T2R-mediated immune activation is inhibited. AMP, antimicrobial peptides; ASL, airway surface liquid; Ca^2+^, calcium ion; Gαgust, Gαgustducin; IP3, inositol trisphosphate; NO, nitric oxide; NOS, nitric oxide synthase; PLCβ2, phospholipase C isoform β2; T1R, sweet taste receptor; T2R, bitter taste receptor; TRPM5, transient receptor potential cation channel subfamily M member ion channel.

Taste receptors are G-protein-coupled receptors that trigger a downstream signaling cascade in response to activation by a specific ligand (Zhang et al., 2003). Initially described in type II cells of the tongue and named for their role in taste (Margolskee, 2002), bitter (T2R) and sweet (T1R) taste receptors are also expressed in a variety of extra-oral tissues (Behrens and Meyerhof, 2010), including the sinonasal cavity (Lee et al., 2012, 2014) and respiratory tract (Shah et al., 2009), and have been recognized in recent years for their essential role in airway immune defense (Tizzano et al., 2010; Lee et al., 2012). More specifically, taste receptors in the upper airway have been described in both ciliated cells (Lee et al., 2012; Lee and Cohen, 2013) and the more rare solitary chemosensory cells (Braun et al., 2011; Barham et al., 2013; Lee et al., 2014; Figure 1).

### Bitter taste receptors in ciliated airway cells

Initial support for the role of the T2R family of bitter taste receptors in upper airway immunity came from a 2009 study characterizing their expression and function in human bronchial ciliated epithelial cells. Researchers demonstrated that T2Rs located on motile cilia increased ciliary beating upon binding of known bitter agonists, establishing a role for cilia as “chemosensory organelles” and launching the hypothesis that T2Rs may detect noxious substances (Shah et al., 2009). However, ligands expected to be found in the airway with physiologic relevance had yet to be identified.

Further evidence for a role of taste receptors in airway immunity came from characterization of the T2R38 isoform in sinonasal ciliated epithelial cells and identification of bacterial acyl-homoserine lactones (AHLs). AHLs are quorum-sensing molecules produced by *Pseudomonas aeruginosa* and other gram negative bacteria and they serve as physiologic T2R38 ligands. Activation of T2R38 by two major *P. aeruginosa* AHLs results in a calcium-dependent increase in nitric oxide (NO) which is both directly bactericidal and also increases ciliary beat frequency and MCC (Lee et al., 2012). As many lactone compounds are bitter (Chadwick et al., 2013), it is logical to hypothesize that they may stimulate T2Rs. However, until Lee et al.'s initial T2R38 study, evidence of AHLs as physiologic ligands was limited to mouse studies. In those studies, stimulation of nasal solitary chemosensory cells (SCCs), which express both bitter and sweet taste receptors, caused a calcium-dependent release of acetylcholine and subsequent trigeminal activation that resulted in breath-holding and inflammatory mediator release (Finger et al., 2003; Tizzano et al., 2010, 2011; Saunders et al., 2014).

Recent research has expanded our understanding of the immune role of bitter taste receptors. T2R38 detection of AHLs has also been described in other cell types including human neutrophils (Maurer et al., 2015), and additional human pathogens such as *Bacillus cereus* have also been found to elicit a T2R-mediated NO response (Workman et al., 2017b). Building on the initial T2R38 studies, more recent studies in primary human sinonasal cultures demonstrated the ubiquitous expression of three other T2Rs, T2R4, and T2R14, T2R16, throughout the sinonasal tract with a similar ability to stimulate NO production in response to their respective ligands (Hariri et al., 2017b; Yan et al., 2017). Further studies are needed to determine the clinical relevance of these other T2R isoforms and their contributions to mucosal defenses.

### T2R bitter and T1R sweet receptors in solitary chemosensory cells (SCCs)

Taste receptor expression in the upper airway is not limited to ciliated cells. Representing approximately 1 in 100 cells, SCCs are discrete non-ciliated cells that express both T2R bitter and T1R2/3 sweet taste receptors as well as other canonical taste signaling proteins (Finger et al., 2003; Tizzano et al., 2011; Barham et al., 2013). They were initially discovered in humans in the vomeronasal epithelium (Braun et al., 2011) and later throughout the sinonasal tract (Lee et al., 2014) at low density (Saunders et al., 2014). Preliminary studies in humans to elucidate SCC physiology with known bitter and sweet agonists found that these two modalities of taste work antagonistically to regulate the immune response. While T2R activation by bitter compounds initiated a traditional calcium taste-signaling cascade described in ciliated cells, T1R2/3 activation by glucose blocked this response (Lee et al., 2014). This regulation of bitter taste receptor signaling by the T1R sweet receptor suggests the ability to inhibit excessive immune activation during low levels of colonization and physiologic airway glucose concentration, and to release inhibition of the T2R-mediated immune response as glucose levels fall in early bacterial infection.

Notably, the downstream effects of T2R stimulation differ between cell types in the upper airway. In SCCs, T2R stimulation does not initiate NO secretion as in ciliated cells but rather propagation of the calcium signal results in rapid release of antimicrobial peptides from neighboring cells (Lee et al., 2014). This response occurs in minutes (Lee et al., 2014), providing support for taste receptors as early detectors of inhaled pathogens and activators of a more rapid immune response. While the T2R-initiated calcium cascade is similar across cell types in the human nasal cavity as well as consistent with earlier studies of SCCs in mice, its role in local immune regulation is distinct from that in ciliated airway cells and in contrast to the downstream effects of trigeminal activation seen in mice, suggesting possible adaptation in function or additional, yet unidentified triggers. Further studies are needed to characterize the role of SCCs and species differences in sinonasal physiology.

## Taste receptor polymorphisms

Taste receptors display marked genetic diversity as evidenced by not only multiple receptor isoforms but also polymorphisms that modulate receptor function (Chandrashekar et al., 2000; Margolskee, 2002) with implications for taste, immunity, and consequently, disease. T2R and T1R polymorphisms correlate with bitter (Hayes et al., 2011) and sweet taste preferences (Mennella et al., 2005; Fushan et al., 2009; Bachmanov et al., 2014) as specific genotypes regulate receptor sensitivity, resulting in absent to intense taste perception of the same compounds between individuals (Bufe et al., 2005). Some of these same polymorphisms also correlate with severity of disease in CRS. Perhaps most well-studied are the polymorphisms in *TAS2R38* that present as functional (PAV) and nonfunctional (AVI) T2R38 variants (Bufe et al., 2005). Compared with homozygotes for the functional allele, patients who are AVI/AVI homozygotes not only cannot taste bitter T2R38 agonists such as PTC but also show a decreased NO response to AHLs (Lee et al., 2012), increased susceptibility to gram-negative upper respiratory infections (Lee et al., 2012) and a higher burden of biofilm formation (Adappa et al., 2016b). Furthermore, AVI/AVI homozygotes with CRS are more likely to require surgical intervention (Adappa et al., 2013, 2014) and a subset of patients with CRS without nasal polyps may have worse outcomes after surgery (Adappa et al., 2016a). While less extensively studied, allele variations in *TAS1R* genes encoding sweet T1R receptors also show significant frequency differences between patients with CRS and controls (Mfuna Endam et al., 2014).

These findings suggest that genotypic variation results in phenotypic differences in both taste perception as well as airway disease. Current research in the field is focused on utilizing this relationship to assess taste receptor variation and function through simple taste tests. Preliminary studies have demonstrated phenotypic taste test differences between CRS and control patients to a variety of bitter and sweet compounds. Patients with CRS without nasal polyps were found to be less sensitive to the bitter compound denatonium while all CRS patients exhibited a hypersensitivity to sucrose (Workman et al., 2017a). This initial data supports phenotypic taste testing as a reflection of disease state. Given the clinical implications of the genotype-phenotype-disease correlation, taste testing to phenotypically assess a patient's taste receptor function and by extension susceptibility to disease or likelihood to benefit from certain treatments appears to be a promising future direction. Further studies are needed to clinically validate compounds and concentrations used to develop a taste test with predictive value.

## Stimulators of bitter and sweet taste receptors and potential therapeutics

The previous studies discussed established the role of taste receptors as upper airway sensors capable of detecting bacteria and stimulating the innate immune response and offered the possibility that taste receptors may have utility as therapeutic targets. However, the genetic diversity of T2Rs and the high frequency of non-functional polymorphisms (Bufe et al., 2005; Lee et al., 2012) would limit their efficacy in this subset of patients. Recent studies have expanded our understanding of the wide range of biologically active compounds that can activate taste receptors and thus modulate upper airway immune defenses (Table 1). This has implications for both pathophysiology as well as treatment as we consider the question of whether pathogens may hijack these systems to circumvent immune detection as well as the possibility of novel therapeutics that could amplify the innate immune response.

**Table 1:** Taste receptor ligands in human sinonasal tissue.

**Airway cell type**	**Taste receptor**	**Chemical compound**	**Endogenous ligand**	**References**
Ciliated Cells^a^	T2R38	Phenylthiocarbamide Propylthiouracil	AHLs (Gram negative bacteria) *N*-butyryl-L-homoserine lactone *C*-6-homoserine lactone *N*-3-oxo-dodecanoyl-L-homoserine lactone	Bufe et al., 2005; Kim and Drayna, 2005; Lee et al., 2012
	T2R4	Colchicine Dapsone	Unknown	Shah et al., 2009; Yan et al., 2017
	T2R14	Plant flavones	Unknown	Hariri et al., 2017b
	T2R16	D-salicin Phenyl β-D-glucopyranoside	Unknown	Yan et al., 2017
SCCs	T1R2/3	Glucose Sucrose Sucralose	Airway surface liquid glucose D-amino acids (*Staphylococcus)*	Jiang et al., 2005; Lee et al., 2014, 2017
	T2R10, T2R46, T2R47/30	Denatonium Absinthin	Unknown	Meyerhof et al., 2010; Wiener et al., 2012; Lee et al., 2014

a *Ciliated cells have also shown a partially T2R-mediated NO response to a compound produced by B. Cereus (Carey et al., 2017). Further purification is needed to identify this compound*.

### Bitter T2R on ciliated cells are activated by flavones

Many of the initial experiments characterizing T2R physiology used canonical bitter compounds that serve as ligands specific to the isoform being studied, for instance phenylthiocarbamide (PTC) activation of T2R38 (Kim and Drayna, 2005) and colchicine and D-Salicin for T2R4 and T2R16, respectively (Yan et al., 2017). The ability of T2Rs to detect bacteria and the chemical compounds they secrete was first described by the activation of T2R38 by AHLs produced by *P. aeruginosa* and the subsequent stimulation of immune mechanisms (Lee et al., 2014; Maurer et al., 2015; Gaida et al., 2016). T2R receptors are thus attractive therapeutic targets to amplify the natural host immune response.

While T2R agonists themselves, such as PTC, could serve as possible therapeutic agents, recent studies have shown promise for a number of biologically active compounds. Flavones, a class of flavonoids produced by plants previously noted to have antibacterial effects, also activate T2Rs (Roland et al., 2011; Kuroda et al., 2016). Studies of the effect of these compounds in the upper airway and on sinonasal pathogens demonstrated their ability to increase the efficacy of innate antimicrobials secreted by ciliated airway epithelial cells on *P. aeruginosa* (Hariri et al., 2017a) and induce NO production and increase MCC through activation of yet another T2R isoform (T2R14) (Hariri et al., 2017b). Identification of other receptor isoforms and the compounds to which they respond is therefore of particular clinical significance for their potential to activate endogenous immune defenses in individuals harboring a polymorphisms that renders T2R38 non-functional.

### Sweet T1R on SCCs are activated by glucose, D-amino acids

Sweet T1R receptors on SCCs have been shown to block immune activation until infection depletes airways surface liquid glucose concentrations, decreasing T1R signaling and releasing its inhibition of T2R-mediated immune responses. The data to support this is strong. Addition of both the bitter compound denatonium and the T1R2/3 agonists glucose and sucrose to sinonasal airway cultures showed inhibition of the T2R-mediated calcium cascade while cultures derived from mice that do not express the T1R2/3 receptors (Lemon and Margolskee, 2009) respond normally (Lee et al., 2014). Furthermore, researchers were able to block glucose/T1R-mediated downregulation of the SCC calcium cascade by using T1R2/3 antagonists such as lactisole (Jiang et al., 2005) and amiloride (Imada et al., 2010; Lee et al., 2014). This has implications not only for our understanding of CRS pathophysiology, as CRS patients have been noted to have chronically elevated ASL glucose (Lee et al., 2014) which may contribute to a cycle of weak immune defenses and chronic infection, but also for potential therapeutic targets.

Interestingly, bacteria may have found a way to co-opt this natural regulatory mechanism. Recent research has shown that D-amino acids produced by *Staphylococcus* species are capable of activating SCC sweet taste receptors and inhibiting T2R-mediated signaling (Lee et al., 2017). While *Staphylococcus* species are frequent sinonasal colonizers, suggesting that this may be one mechanism that allows them to evade immune eradication, T1R antagonists could be useful therapies in *Staphylococcus*-driven infections. Pharmacologic suppression of this T1R downregulatory pathway could thus prove to be a useful adjunct in augmenting the immune response, both in patients who would not be expected to respond to bitter agonists, but also in infection with particular bacterial species that have hijacked this pathway to evade immune detection. However, D-amino acids also inhibited *P. aeruginosa* biofilm formation, suggesting that immune suppression allowing commensal bacterial growth may offer protection against more pathogenic bacterial strains. Further research is needed to clarify the nature of such bacterial-host interactions prior to moving forward with T1Rs as a therapeutic target.

## Conclusions

There is significant evidence to support the function of taste receptors as regulators of upper airway immunity. Activation of these receptors results in immediate-onset immune activation, complementing the more sustained and slower response achieved by activation of traditional innate immunity pathways such as TLRs, or at times suppression of the innate immune response. A wide range of biologically active compounds that activate T1R and T2R receptors have recently been described, increasing our understanding of not only additional receptor isoforms but also how receptor function may contribute to the pathophysiology of CRS. These findings suggest that targeting T1R and T2R receptors may allow for augmentation of the airway immune response and identify novel therapeutic targets that would allow the future treatment of CRS to be tailored to an individual patient's receptor profile. While further testing is needed, simple taste tests to determine taste perception phenotypes, which have shown a correlation with taste receptor genotype, may be used to predict not only susceptibility to disease but also potential response to a variety of therapies, allowing for truly personalized treatment.

## Author contributions

VT performed the literature search and wrote the manuscript. All authors reviewed and approved of the final manuscript.

### Conflict of interest statement

The authors declare that the research was conducted in the absence of any commercial or financial relationships that could be construed as a potential conflict of interest. The reviewer RJ and handling Editor declared their shared affiliation.
